# Cold exposure induces the constitutively active thermogenic receptor, GPR3, via ERRα and ERRγ

**DOI:** 10.1016/j.molmet.2025.102277

**Published:** 2025-10-30

**Authors:** Olivia Sveidahl Johansen, Rebecca L. McIntyre, Janane F. Rahbani, Qiaoqiao Zhang, Charlotte Scholtes, Damien Marc Lagarde, Cyrielle Billon, Isabelle Côté, Maria Delgado-Martin, David Tandio, Astrid Linde Basse, Elodie Eury, Anastasia Kralli, Thomas P. Burris, Vincent Giguère, Lawrence Kazak, Zachary Gerhart-Hines

**Affiliations:** 1 Novo Nordisk Foundation Center for Basic Metabolic Research, University of Copenhagen, Blegdamsvej 3B, 2200 Copenhagen, Denmark; 2 Center for Adipocyte Signaling, University of Southern Denmark, Campusvej 55, 5230 Odense, Denmark; 3 Section of Hematology and Oncology, Department of Medicine, The University of Chicago, Chicago, IL 60637, USA; 4 Rosalind & Morris Goodman Cancer Institute and Department of Biochemistry, McGill University, 3655 Promenade Sir William Osler, Montreal, QC, H3G 1Y6, Canada; 5 Laboratoire CarMeN, UMR INSERM U1060/INRA U1397, Université Claude Bernard Lyon 1, F-69310 Pierre-Bénite and F-69500 Bron, France; 6 Department of Pharmaceutical and Administrative Sciences, Center for Clinical Pharmacology, Washington University School of Medicine and St. Louis College of Pharmacy, St. Louis, MO 63110, USA; 7 Department of Cellular and Systems Pharmacology, University of Florida College of Pharmacy and University of Florida Genetics Institute, Gainesville, FL 32610, USA; 8 Department of Physiology, The Johns Hopkins University School of Medicine, Baltimore, MD 21205, USA; 9 Embark Laboratories ApS, Ottiliavej 9, 2500 Valby, Denmark; 10 Incipiam Pharma ApS, Ole Maaløes Vej 3, 2200 Copenhagen, Denmark

**Keywords:** GPR3, ERRα, ERRγ, G protein-coupled receptors, brown adipose tissue, Thermogenesis, Transcriptional regulation

## Abstract

**Objectives:**

Despite transformative advances in obesity pharmacotherapy, safely increasing energy expenditure remains a key unmet need. Exploiting thermogenic adipocytes represents a promising target given their capacity for significant catabolic activity. We previously showed that G protein-coupled receptor 3 (GPR3) can drive energy expenditure in brown and white mouse and human adipocytes. GPR3 is a unique GPCR because it displays high intrinsic activity and leads to constitutive cAMP signaling upon reaching the cell surface. Therefore, the transcriptional induction of GPR3 is analogous to ligand-binding activation of most GPCRs. *Gpr3* expression is physiologically induced in thermogenic adipocytes by cold exposure, and mimicking this event through overexpression in mice is fully sufficient to increase energy expenditure and counteract metabolic disease. Yet the factors mediating physiological *Gpr3* expression remain unknown.

**Methods:**

Here, we apply ATAC-Seq to identify cold-induced promoter elements of *Gpr3*. We uncover a role for the estrogen-related receptors, ERRα and ERRγ, in the physiological transcriptional control of *Gpr3* using adipose-specific double knock-out mice with and without adeno-associated virus (AAV)-mediated rescue.

**Results:**

We show that ERRα directly binds the cold-induced promoter element of *Gpr3* and that ERRα, ERRβ, and ERRγ each activate the *Gpr3* promoter *in vitro* when co-transfected with PGC-1α. Adipocyte ERRα and ERRγ are required for the *in vivo* transcriptional induction of *Gpr3* during cold exposure. Importantly, deficient *Gpr3* cold-inducibility in adipose-specific ERRα and ERRγ KO mice is fully rescued by delivery of AAVs re-expressing either ERRα or ERRγ directly into brown adipose tissue.

**Conclusions:**

ERRα and ERRγ are critical regulators of cold-induced transcription of *Gpr3* and represent a targetable strategy for pharmacologically unlocking GPR3-induced energy expenditure.

## Highlights

• The *Gpr3* promoter contains differentially accessible ERR elements in BAT in mice exposed to cold.
• ERRα binds to the *Gpr3* promoter.
• Cold-inducibility of *Gpr3* is fully dependent on ERRα and ERRγ *in vivo.*

## 1 Introduction

Thermogenic adipocytes have a unique capacity for utilizing metabolites and lipids from the bloodstream to generate heat through catabolic processes [[1], [2], [3], [4]]. These macronutrient-consuming and energy-dissipating activities are linked to cardiometabolic protection and enhanced glycemic control in humans [[5], [6], [7], [8], [9], [10], [11]], making thermogenic adipocyte activation an appealing strategy for treatment of metabolic disorders like obesity and diabetes. The activity of thermogenic adipocytes is highly orchestrated by G protein-coupled receptors (GPCRs) [12,13], with the Gα_s_-coupled β-adrenergic receptors serving as primary regulators of adipocyte catabolic recruitment. GPCRs represent the most druggable receptor class in biology [[14], [15], [16]], and therefore understanding the various GPCRs that control thermogenic adipose and their regulation is of significant interest for therapeutic purposes [13]. We and others recently discovered GPR3 as a potent activator of mouse and human thermogenic adipocytes [17,18]. GPR3 confers constitutive Gα_s_-coupled receptor signaling due to innate structural features of the receptor as well as a cell autonomously produced lipid ligand [17,[19], [20], [21]]. As a result, genetic or viral overexpression of GPR3 is alone sufficient to promote the thermogenic activity of adipocytes *in vitro* and *in vivo* and improve parameters of metabolic health [17]. This potential for cardiometabolic disease therapy has contributed to more recent focus on developing synthetic GPR3 agonists [22]. Given that GPR3 begins increasing cAMP production as soon as it reaches the cell surface [23], the transcriptional control of *Gpr3* represents a key point of regulation. Therefore, transcriptional induction of GPR3 represents a stimulatory event comparable to the ligand-induced activation of more canonical GPCRs. We previously showed that environmental cold exposure acutely induces GPR3 transcription through a lipolytic signal [17] however, the transcriptional machinery mediating GPR3 activation remains unknown.

The estrogen-related receptors (ERRs) are a family of orphan nuclear receptor transcription factors that share sequence similarities with the estrogen receptors [24] and yet regulate distinct gene programs [25]. The three known isoforms of ERRs – ERRα, ERRβ, and ERRγ – are widely implicated in the control of cellular energy metabolism, including glucose and lipid homeostasis and mitochondrial biogenesis [[26], [27], [28]]. The ERRs are constitutively active [29,30]. However, protein coactivators, in particular peroxisome proliferator-activated receptor γ coactivator 1α (PGC-1α) and peroxisome proliferator-activated receptor γ coactivator 1β (PGC-1β), significantly impact their regulatory activity [28,[30], [31], [32]].

Critical roles of ERRα and ERRγ (together designated as ERRα/γ) in liver and muscle metabolism are well-established [28,[33], [34], [35]]. Moreover, recent advances have underscored the important roles of these nuclear receptors in the adrenergic-driven remodeling of brown adipose tissue (BAT) [[36], [37], [38]]. Whole-body ERRα KO mice are sensitive to cold exposure and display reduced BAT mitochondrial content [37]. The transcriptional remodeling upon β3-adrenergic stimulation in BAT is highly dependent on ERRα/γ and loss of adipocyte ERRα/γ impairs BAT thermogenic capacity [38]. The ERRs are more highly expressed in BAT than in white adipose tissue (WAT), with ERRα being the most prevalent, followed by ERRγ and then ERRβ [39,40]. The coactivator PGC-1α (encoded by *Ppargc1a*) is also enriched in BAT and highly induced upon cold exposure [41]. Finally, the ERRs are proposed targets for activation in the treatment of metabolic diseases [28,38,42]. Recent optimization of synthetic pan-ERR agonists with pharmacokinetic properties that allow *in vivo* testing suggest that targeting ERRs may be a pharmacologically viable strategy [43,44].

In the present study, we investigate the chromatin dynamics surrounding the *Gpr3* transcriptional start site (TSS) in response to cold exposure, when *Gpr3* is transcriptionally activated. We identify a cold-responsive promoter element of *Gpr3* and show that ERRα is enriched at the promoter. We establish that all three ERRs can activate the *Gpr3* promoter *in vitro*, then confirm that ERRα/γ are required for physiological *Gpr3* cold induction through viral targeted rescue in tissue specific KO mice.

## 2 Materials & methods

### 2.1 HEK293T cell culture

HEK293T cells (ATCC, 293T) were cultured in DMEM (Wisent, 319-005-CL) supplemented with 10% FBS (Multicell, 098150) and 1% penicillin/streptomycin (yielding ∼1.79 μM penicillin, and ∼17.2 μM streptomycin, Multicell, 450-201-EL). The cells were maintained at 37 °C in a humidified atmosphere with 5% CO_2_.

### 2.2 Luciferase assay

HEK293T cells were seeded on white 96-well plates 24 h prior to transfection to achieve ∼80% confluence at time of transfection.

Custom vectors were obtained from VectorBuilder. The *Gpr3* promoter luciferase construct included the sequence of the *Gpr3* proximal promoter (defined as 4 kb upstream of the transcription start site) inserted into a pGL4.10[Luc2] vector. For the ERRE deletion construct, base pairs 2555–2700 (surrounding the proposed ERREs) were excised from *Gpr3* promoter luciferase construct.

Cells were transfected with 50 ng of the firefly luciferase reporter construct along with 50 ng of each of the indicated pcDNA expression plasmids using the TransIT-X2 Dynamic Delivery System (Mirus Bio) according to the manufacturer's instructions (1 μL per well). To normalize for transfection efficiency, 0.01 ng of a NanoLuc luciferase control plasmid (pNL1.1[Nluc]) was included in each condition. Plasmids and transfection reagent were diluted in Opti-MEM reduced serum medium (Gibco). The total DNA amount per well was kept constant by addition of empty pcDNA vector when necessary.

At 24 h post-transfection, media was changed, and cells were treated with 10 μM isoproterenol or water vehicle. After 5 h incubation, Firefly and NanoLuc luciferase activities were measured sequentially using the Nano-Glo Dual-Luciferase Reporter Assay (Promega) on a CLARIOstar Plus Microplate Reader (BMG Labtech). Raw luminescence values were recorded, and firefly luciferase activity was normalized to the corresponding NanoLuc luminescence (Firefly/NanoLuc ratio). Data are expressed relative to cells transfected with reporter plus empty pcDNA vector set as 1.0.

### 2.3 Animals

#### 2.3.1 ERRa/y KO mice

The mouse studies performed adhered to the guidelines established by the Canadian Council of Animal Care and were approved by the Animal Resource Centre at McGill University.

The mice were kept on a 12-hour light and 12-hour dark schedule with lights on at 07:00 h. The mice had access to a low-fat diet (2920X, Envigo) and drinking water *ad libitum*. They were maintained in an enriched environment with bedding and shredded paper strips and housed in groups of 3–5 per cage at 22 °C ± 2 °C, until they were 7–12 weeks old, when experiments were initiated. During the TN (30 °C) acclimation and cold exposure (4–6 °C) experiments, the mice were housed in separate cages with bedding and shredded paper strips. Experiments were conducted on age-matched littermates at the specified temperatures. The mice were euthanized by cervical dislocation, and interscapular BATs were snap-frozen in liquid nitrogen and preserved at −80 °C for subsequent analysis. AdipoQ-Cre *Esrra/g* KO mice were obtained by breeding AdipoQ-Cre (B6.FVB-Tg(AdipoQ-Cre)1Evdr/J: JAX stock #028020 [45]) mice maintained on a C57BL/6J background to ERRα/γ^fl/fl^ mice [38,[46], [47], [48]]. The sexes of the mice used for experiments are indicated in the figure legends. Genotyping primer sequences are available in Supplementary Table 3.

#### 2.3.2 WT mice

A cohort of two-month-old-C57BL/6 male mice obtained from Jackson Laboratories (Bar Harbor, ME) was maintained under a 12:12 light cycle at ambient temperatures (22–25 °C) and had free access to drinking water and chow diet (2018 Teklad Global 18% Protein Rodent Diets, Inotiv, Lafayette, IN). These studies were conducted at the University of Florida and Washington University in accordance with procedures approved by the Institutional Animal Care and Use Committee.

### 2.4 Power calculation for AAV-mediated rescue study

The primary experimental output was *Gpr3* induction during 24 h cold exposure. Therefore, group size was determined using R, based on *Gpr3* expression in BAT following a pilot 24 h cold exposure study. Output from R determined a minimum group size of 2.73, so at least 3 mice were included per group.

Male and female mice were included in the study. Male mice are designated in figures as triangle points, while females are designated as circle points. Experiments with male and female mice were conducted independently but are pooled in measures of gene expression. No robust sex-dependent differences were observed.

### 2.5 Site-directed adeno-associated virus (AAV) delivery

The AAV backbone for generation of AAV plasmids was kindly provided by the lab of Professor Christian Wolfrum (ETH Zurich). 293T cells (ATCC) were seeded at 80–90% confluence in a 15-cm cell culture plate. Immediately before transfection, media was replaced with 20 ml DMEM (Wisent, 319-005-CL) supplemented with 10% FBS (Multicell, 098150) and 1% penicillin/streptomycin (yielding ∼1.79 μM penicillin, and ∼17.2 μM streptomycin, Multicell, 450-201-EL). 10 μg of targeting vector plasmid and 39.5 μg of helper plasmid pDP8 (Plasmid Factory, PF421-180518) were mixed in 2.5 ml Optimem (Gibco, 31985) before addition of 200 μl PEI (1 mg/ml) (Polysciences, 23966-1). The transfection mix was briefly vortexed and incubated for 10 min at RT. Then, the transfection mix was added dropwise onto the cells. The culture medium was refreshed 24 h after the transfection. Culture medium was then collected 72 h after transfection, and virus was concentrated using AAVanced Concentration Reagent (System Biosciences, AAV100A-1) in accordance with the manufacturer's guidelines.

Site-directed delivery of AAVs into BAT was carried out as previously described [49]. The mice were anaesthetized by isoflurane at a concentration of 2.5% for induction and 1.5% for maintenance, and local (Lurocaine, Vetoquinol and Bupivacaine injection BP, Sensorcaine in 1:1 ratio) and general (Carprofen, 5 mg per kg body weight) (Rimadyl injectable solution, Zoetis Canada) analgesia were administered. Body temperature was maintained using a heating pad. To expose the brown fat depot, a longitudinal incision of 0.5–1.0 cm was made at the interscapular region. Using a 10 ml Hamilton syringe, 20 μl of AAV (≈10^12^ vg/ml) were administered across multiple (at least 5) injections within each lobe of the interscapular brown fat depot. This approach was employed to achieve a more homogeneous and extensive viral spread throughout the tissue. The incisions were closed by surgical clips and surgical glue (3M Vetbond™^/TC^). The mice were monitored daily and received general analgesia by subcutaneous injections on days 1 and 2 post-surgery. 9 days post-surgery, the mice were single-housed, and the surgical clips were removed.

### 2.6 Cold exposure

Mice were allowed 5 days to acclimate to thermoneutrality (TN, 30 °C) before the temperature challenges. Cold challenges ranged from 4 to 6 °C and specific durations are listed in the figure legends.

### 2.7 Pharmacological intervention and analysis

C57BL/6 male mice (n = 3/group) were intraperitoneally administered SLU-PP-915 (20 mg/kg; b.i.d. with 10 ± 2 h between doses) or vehicle (DMSO, Tween 80, PBS; 10/10/80; %vol/%vol/%vol; 10 mL/kg) for 5 days. Interscapular brown adipose tissue was collected and snap frozen until tissue processing.

### 2.8 RNA extraction

RNA was extracted from snap-frozen interscapular BAT and subcutaneous WAT samples using QIAzol (Qiagen, 79306) and purified with RNeasy Mini spin columns (Qiagen, 74104) according to the manufacturer's instructions. RNA was quantified using a NanoDrop 8000 Spectrophotometer (Thermo Scientific Pierce) prior to cDNA synthesis.

### 2.9 RT–qPCR

RNA was reverse transcribed using a High-Capacity cDNA Reverse Transcription kit (Applied Biosystems, 4368813). 10 ng cDNA and 150 nmol of each primer were mixed with 3 μl GoTaq qPCR Master Mix (Promega, A6001) to a final volume of 6 μl per sample. RT–qPCRs were performed using a CFX384 real-time PCR system (Bio-Rad) in a 384-well format, using the following cycling conditions: one step at 95 °C for 3 min, then 95 °C for 10 s, 60 °C for 20 s, 72 °C for 10 s. Expression data were quantified by ΔCt calculations normalized to housekeeping genes *36b4* (*Rplp0*) or *Ppib*.

### 2.10 Western blots

Samples were homogenized in lysis buffer (50 mM Tris, pH 7.4, 500 mM NaCl, 1% NP40, 20% glycerol, 5 mM EDTA, and 1 mM PMSF), supplemented with Roche protease inhibitors (Roche, 11836170001) using the TissueLyser II for 10 min at 20 Hz. The homogenates were cleared by centrifugation at 20,000 g for 10 min at 4 °C. Protein concentrations were determined using a bicinchoninic acid assay (Pierce, 23225) according to the manufacturer's protocol. Solutions containing 10 μg of protein lysates were denatured in Laemmli buffer (Bio-Rad, 161–0747) supplemented with 10% β-mercaptoethanol and incubated at 95 °C for 10 min. The samples were resolved by 10% Tris/Glycine SDS–PAGE and transferred to a polyvinylidene difluoride membrane. Following the transfer, the membranes were blocked for 1 h in TBS containing 0.05% tween 20 (TBS-T) and 5% milk powder. The membranes were washed 3 times for 10 min in TBS-T prior to incubation with primary antibodies (antibody dilutions are stated in Supplementary Table 2). Primary antibodies were diluted in TBS-T containing 5% BSA and 0.02% sodium azide. The membranes were incubated with primary antibodies at 4 °C overnight. On the following day, the membranes were washed 3 times for 10 min in TBS-T prior to incubation with secondary antibodies. Secondary antibodies were diluted in TBS-T containing 5% milk powder. Membranes were washed 3 times for 10 min before visualization with enhanced chemiluminescence western blotting substrates (Bio-Rad, 1705060). Western blot images were quantified using ImageJ [50], measuring the integrated density of each band and normalizing that value to the integrated density of the membrane-cytoskeletal protein vinculin (VCL) that was used as the loading control.

### 2.11 ChIP-qPCR

ChIP-qPCR was performed as previously described [48]. Nuclei were isolated from 3 interscapular BAT pads per condition, each from individual WT mice. BAT pads were stroked in a nuclei preparation buffer (10 mM HEPES pH7.5, 10 mM KCl, 1.5 mM MgCl2, and 0.1% NP40), 25X with pestle A and 15X with pestle B, and the solution was filtered through a 100 μm strainer. Next, the nuclei were fixed with formaldehyde (1% final) for 12 min at RT, quenched by 125 mM of glycine for 10 min, and washed twice with PBS/0.1% NP40. Subsequently, chromatin was sonicated in 1 ml of sonication buffer (50 mM Tris–HCl pH 8.1, 10 mM EDTA, and 1% SDS) to obtain fragments of around 500 bp. 20 μg chromatin DNA was diluted in ChIP dilution buffer (16.7 mM Tris–HCl pH 8.1, 1.1% Triton X-100, 167 mM NaCl, 1.2 mM EDTA, and 0.01% SDS) up to 2 ml. 10 μl of anti-ERRα antibody (Abcam, ab76228) were added to the sonicated chromatin and left to rotate overnight at 4 °C. The next day, 50 μl of Dynabeads protein G (Thermo Fisher Scientific, 10009D) were washed twice with PBS containing 0.5% tween and 0.5% BSA and added to the chromatin for 1 h under rotation at 4 °C. Next, beads were washed twice with 1 ml of cold low salt RIPA buffer (20 mM Tris–HCl pH 8.1, 0.1% SDS, 1% Triton x-100, 1 mM EDTA, 140 mM NaCl, and 0.1% Na-deoxylcholate), twice with 1 ml of cold high salt RIPA Buffer (20 mM Tris–HCl pH 8.1, 0.1% SDS, 1% Triton x-100, 1 mM EDTA, 500 mM NaCl, and 0.1% Na-deoxylcholate), twice with 1 ml of cold LiCl wash buffer (10 mM Tris–HCl pH 8.1, 250 mM LiCl, 0.5% NP40, 0.5% Na Deoxycholate, and 1 mM EDTA), and twice with RT TE buffer (10 mM Tris–HCl pH 8.0 and 1 mM EDTA). DNA was eluted overnight at 65 °C with 100 μl of ChIP elution buffer (10 mM Tris–HCl pH 8.0, 5 mM EDTA, 300 mM NaCl, 0.1% SDS, and 5 mM DTT) and 16 μl reverse cross-linking mix (250 mM Tris–HCl pH 6.5, 62.5 mM EDTA pH 8.0, 1.25 M NaCl, 5 mg/ml Proteinase K, and 62.5 ug/ml RNAse A) were added. Finally, chromatin immunoprecipitated DNA was purified using a QIAquick PCR purification kit and eluted in 31 μl of elution buffer (10 mM Tris–HCl pH 8.0 and 0.1 mM EDTA). Relative ChIP fold enrichments were controlled by inputs and normalized to the average of two non-specific control regions using a LightCycler 480 (Roche) and SYBR Green I Master Mix (Roche, 4887352001) as previously published [48]. Gene-specific primers used for ChIP-qPCR analysis are listed in Supplementary Table 3. Results represent the average of 3 biological replicates.

### 2.12 ATAC-Seq

Data was extracted from a published dataset [48] and analyzed as previously described [48].

### 2.13 Statistical analyses

Statistical analyses were performed using GraphPad Prism 9. Applied statistical analyses are stated in the figure legends.

## 3 Results

**Figure 1:**
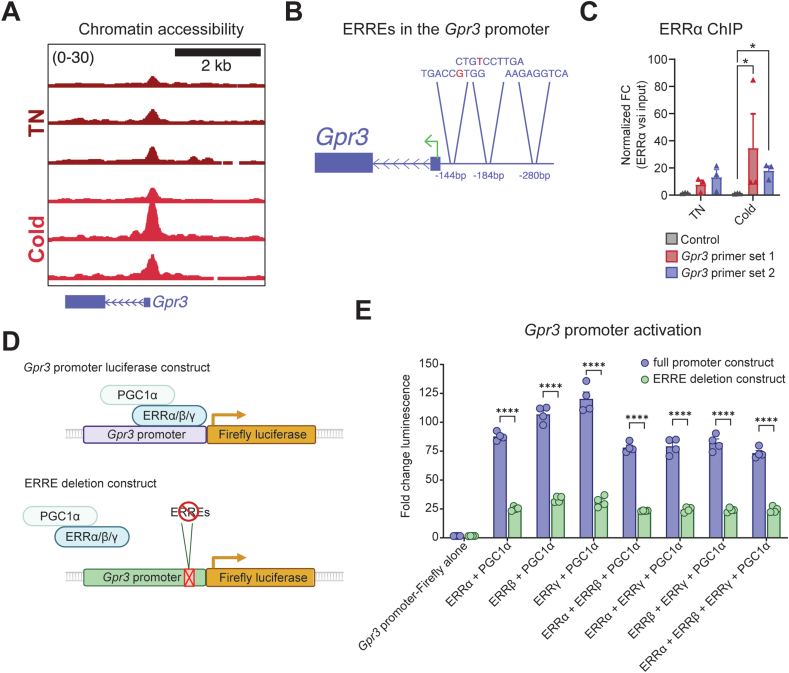
Identification of ERRs as potential regulators of *Gpr3* cold induction (A) ATAC-Seq tracks showing cold-induced differentially accessible region (DAR) upstream of *Gpr3*. BAT nuclei were isolated from mice housed at thermoneutrality (TN) and following 24 h cold exposure (n = 3 per group, males). Data obtained from Rahbani et al. [48]. (B) Potential ERREs located in the identified DAR. Red letters indicate nucleotides aberrant from known ERRE variants [25,[70], [71], [72], [73]]. (C) ChIP-qPCR of ERRα bound to *Gpr3* DAR. BAT nuclei were isolated from mice treated as in A. Two different primer sets covering the DAR were tested. Data were log_10_ transformed prior to analysis by two-way analysis of variance (ANOVA); post hoc comparison using Šídák's multiple comparisons test. (D) Illustration of *Gpr3* promoter constructs used to measure luminescence in (E). HEK293T cells were co-transfected with a firefly luciferase reporter plasmid linked to the *Gpr3* promoter and plasmids expressing the indicated pcDNA expression vectors, along with a NanoLuc control plasmid for normalization. This activation was then compared to activation with an expression plasmid missing the region containing the ERREs shown in (B). (E) Measures of *Gpr3* promoter activation using luciferase comparing the full promoter plasmid and the ERRE deletion plasmid. HEK293T cells were co-transfected with a firefly luciferase reporter plasmid linked to the *Gpr3* promoter and plasmids expressing the indicated pcDNA expression vectors, along with a NanoLuc control plasmid for normalization. Luciferase activity was assayed 24 h later, and firefly values were normalized to NanoLuc luminescence. Activity is expressed relative to the reporter plus an empty pcDNA vector. Data were analyzed by two-way ANOVA; post hoc comparison using Šídák's multiple comparisons test comparing luminescence of each transfection condition to transfection with the ERRE deletion construct. Data are shown as the mean + SEM; ∗P ≤ 0.05, ∗∗∗∗P ≤ 0.0001.

Adipose *Gpr3* is transcriptionally activated in response to cold exposure [17]. Therefore, we hypothesized that a cold-sensitive transcriptional element may be located within or near the *Gpr3* locus. We reanalyzed a dataset recently published by Rahbani et al. in which transposase-accessible chromatin sequencing (ATAC-Seq) was used to examine BAT nuclei isolated from mice housed at thermoneutrality or exposed to a 24 h cold challenge [48]. Here, we identified a differentially accessible region (DAR) immediately upstream of the *Gpr3* TSS (peak position −121 relative to *Gpr3* TSS, GRCm38/mm10), indicative of a proximal promoter (Figure 1A). The promoter element contained three potential estrogen related receptor elements (ERREs) (Fig. 1B), in line with the notion that the ERRs preferably bind in close proximity to the promoters of genes [28]. The ERRs are enriched in BAT compared to WAT [39,40] and directly regulate expression of genes important for oxidative and thermogenic processes [28,37,38,40,[51], [52], [53]]. Collectively, this prompted us to explore a potential role of the ERRs in the transcriptional regulation of *Gpr3*. Because ERRα is more highly expressed in BAT than the other ERR isoforms [39], we assessed ERRα-binding at the promoter region. We performed chromatin immunoprecipitation coupled to quantitative PCR (ChIP–qPCR), testing two sets of primers, each covering the identified ERREs. ERRα was significantly enriched at the promoter compared to a control region that does not bind ERRα (Fig. 1C). Of note, the binding was independent of housing temperature (Fig. 1C). Thus, cold exposure activates a proximal promoter of *Gpr3*, which is enriched for ERREs that bind ERRα.

Next, we cloned the *Gpr3* promoter upstream of a luciferase reporter. This promoter reporter construct was then co-transfected into HEK293 cells along with plasmids expressing *Esrra*, *Esrrb*, *Essrg*, *Ppargc1a* and *Ppargc1b* to test what combinations of the ERRs and PGCs could activate the *Gpr3* promoter (Fig. 1D). Both ERRα and ERRβ required PGC-1α or PGC-1β coactivation, while ERRγ could activate the promoter independently (Fig. S1). This observation is in line with observations that ERRγ is less dependent on the presence of coactivators [40,54,55]. β-adrenergic stimulation with isoproterenol did not influence activation of the promoter in these conditions (Fig. S1), though this result may be affected by low expression of β-adrenergic receptors in HEK293 cells. To test the necessity of the ERREs identified in the cold-regulated DAR (Fig 1B), we compared activation of the full promoter and a construct deleting the region spanning the three ERREs (Figure 1D–E). The deletion of the ERREs significantly diminished activation of the *Gpr3* promoter in the presence of all combinations of ERRs and PGC-1α (Fig 1E). Therefore, the cold-regulated DAR is necessary for ERR activation of the *Gpr3* promoter.

**Figure 2:**
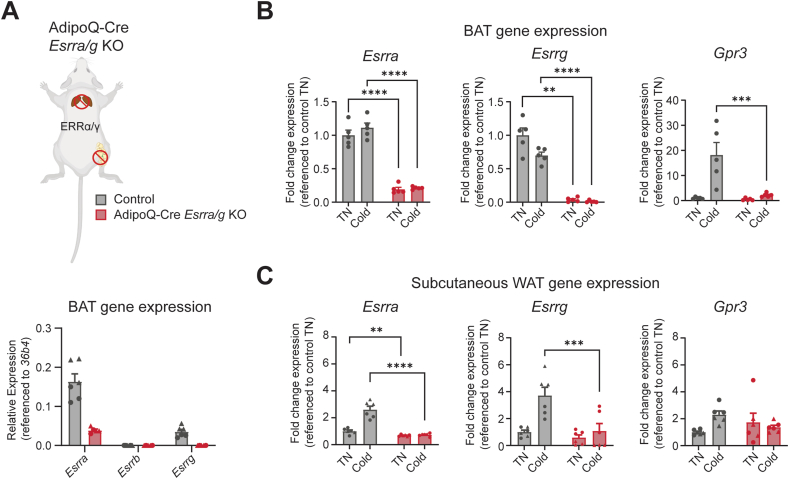
ERRα/γ are required for *Gpr3* cold-inducibility *in vivo* (A) AdipoQ-Cre *Esrra/g* KO mouse model and RT-qPCR measuring gene expression of *Esrra*, *Esrrb* and *Esrrg* in BAT isolated from control and AdipoQ-Cre *Esrra/g* KO mice housed at thermoneutrality (n = 6 per group, males (triangles) and females (circles)). (B) RT-qPCR measuring gene expression of *Esrra*, *Esrrg* and *Gpr3* in BAT isolated from mice housed at thermoneutrality and following 24 h cold exposure (n = 5 per group, females). Data presented as fold change. (C) RT-qPCR measuring gene expression of *Esrra*, *Esrrg* and *Gpr3* from inguinal WAT isolated from mice housed at thermoneutrality and following 24 h cold exposure (n = 6 per group, males (triangles) and females (circles)). RT–qPCR data were log_10_ transformed prior to analysis by two-way ANOVA; post hoc comparison, testing genotype effect at each temperature using Šídák's multiple comparisons test. Data are shown as the mean + SEM; ∗∗P ≤ 0.01, ∗∗∗P ≤ 0.001, ∗∗∗∗P ≤ 0.0001.

We next explored whether the ERRs regulate *Gpr3* transcription *in vivo*. To do so, we utilized an adipocyte-selective *Esrra* and *Esrrg* double knock-out mouse model (AdipoQ-Cre *Esrra/g* KO) (Figure 2A). In BAT, ERRγ (encoded by *Esrrg*) can compensate loss of ERRα (encoded by *Esrra*) [38]. *Esrrb* expression is lower than that of *Esrra* and *Esrry* in control BAT, and significantly decreased upon deletion of *Esrra/g* [38] (Fig. 2A). Thus, the double AdipoQ-Cre *Esrra/g* KO can be considered close to a triple ERR KO in BAT when studying ERR-dependent gene expression.

In both BAT and subcutaneous 10.13039/501100006141WAT, adipocyte-specific ablation of *Esrra* and *Esrrg* significantly blunted the cold induction of *Gpr3* (Figure 2B–C), supporting the hypothesis that these nuclear receptors play a central role in the physiological control of GPR3. While the induction of *Gpr3* is weaker in WAT than in BAT, a longer duration of cold exposure has been shown to increase induction of *Gpr3* in WAT [17].

**Figure 3:**
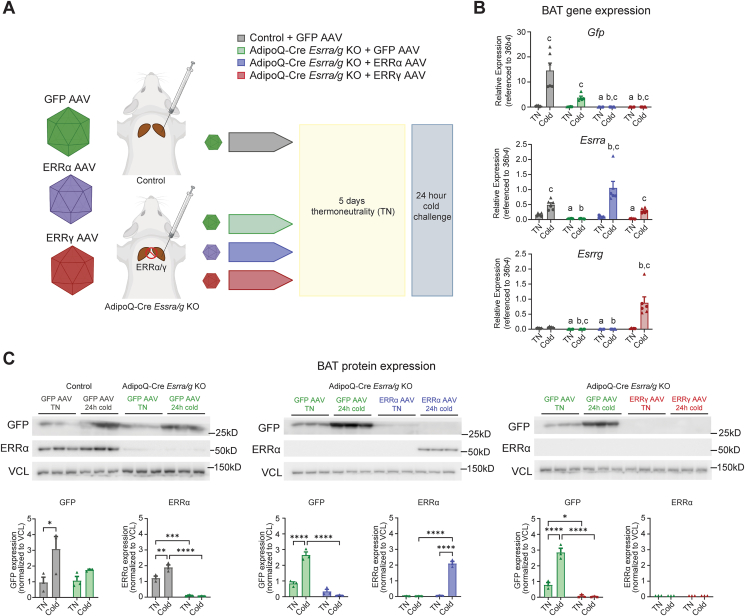
AAV-mediated rescue of *ERRα* and *ERRγ* in AdipoQ-Cre *Esrra/g* KO mice (A) Illustration of AAV-mediated rescue protocol. Site-directed delivery of AAVs expressing *Gfp*, *Esrra*, or *Esrrg* was performed in control and AdipoQ-Cre *Esrra/g* KO mice. After recovery, the mice were housed at thermoneutrality (TN) or challenged with 24 h cold exposure. (B) RT–qPCR from BAT isolated from mice treated as in (A) (n = 5–6 per group, females (circles) and males (triangles). Data points are pooled from two independent experiments). RT–qPCR data were log_10_ transformed prior to analysis by two-way ANOVA; post hoc comparison between control groups and AdipoQ-Cre *Esrra/g* KO groups using Šídák's multiple comparisons test. a = at TN, significantly different from control group, b = at cold, significantly different from control group, c = significantly different between TN and cold within group. (C) Western blot and quantifications from BAT isolated from mice treated as in A (n = 3 per group, males). Western blot quantification data were compared by two-way ANOVA; post hoc comparison between all groups using Tukey's multiple comparisons test. Data are shown as the mean + SEM; ∗∗P ≤ 0.05, ∗∗P ≤ 0.01, ∗∗∗P ≤ 0.001. ∗∗∗∗P ≤ 0.0001.

Mice lacking ERRα/γ display compromised BAT remodeling in response to pharmacological β3-adrenergic agonism [38]. Therefore, the effects we observe on *Gpr3* could be secondary to diminished adipocyte function. To determine if the impaired *Gpr3*-inducibility in this mouse model was directly a result of ERRα/γ loss-of-function, we established an acute gene rescue protocol. We restored ERRα or ERRγ expression in the interscapular BAT depots of the *Esrra/g* KO mice using site-directed delivery of AAVs expressing *Gfp*, *Esrra*, or *Esrrg* under the control of an uncoupling protein 1 (*Ucp1*) promoter (Figure 3A). *Ucp1* is uniquely expressed in thermogenic adipocytes [1]. Thus, the *Ucp1* promoter, in combination with site-directed delivery of AAVs enabled BAT-targeted ERRα/γ rescue. Two weeks after viral delivery (9 days recovery from surgery at RT, then 5 days acclimation to TN), the mice were exposed to a 24 h cold challenge, and BAT depots were isolated for analysis. The rescue was confirmed by qPCR and western blot (Figure 3B–C). In fact, AAV-mediated gene transfer resulted in higher mRNA levels of *Esrra* and *Esrrg* compared to controls after 24 h of cold exposure (Fig. 3B). This is likely due to the cold-inducibility of the *Ucp1*-promoter driving the AAV-mediated overexpression. ERRα expression was only rescued in the cold exposed group (Fig. 3C). We attempted to measure ERRγ protein expression using the only validated specific ERRγ antibody [38], but were unable to detect a clear signal for ERRγ and therefore cannot confirm rescued protein expression.

**Figure 4:**
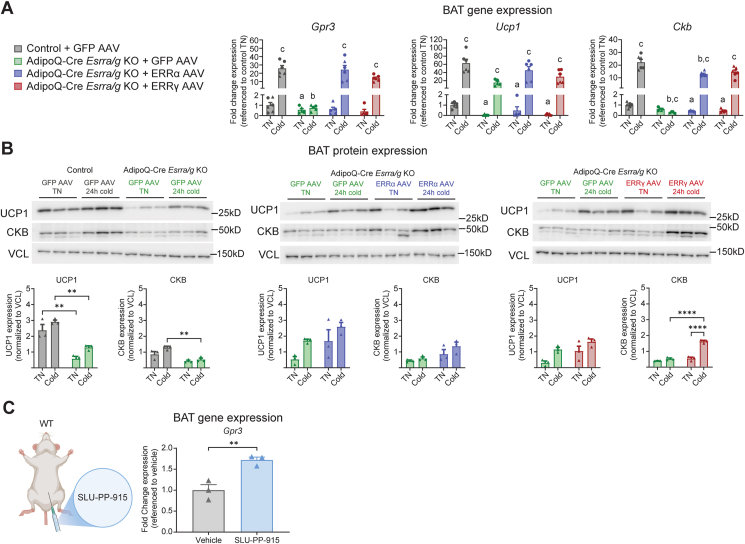
AAV-mediated rescue of *ERRα* and *ERRγ* restores cold-inducibility of *Gpr3* (A) RT–qPCR from BAT isolated from mice treated as in Figure 3A. RT–qPCR data were log_10_ transformed prior to analysis by two-way ANOVA; post hoc comparison between control groups and AdipoQ-Cre *Esrra/g* KO groups and between temperatures within each genotype using Šídák's multiple comparisons test. a = at TN, significantly different from control group. b = at cold, significantly different from control group, c = significantly different between TN and cold within group. Data presented as fold change. (n = 5–6 per group, females (circles) and males (triangles)). (B) Western blot and quantifications from BAT isolated from mice treated as in (B) (n = 3 per group, males). Western blot quantification data were compared by two-way ANOVA; post hoc comparison between all groups using Tukey's multiple comparisons test. Data are shown as the mean + SEM. (C) C57BL/6 mice were treated with SLU-PP-915 (20 mg/kg; b.i.d) or Vehicle (DMSO, Tween 80, PBS; 10/10/80; %vol/%vol/%vol; 10 mL/kg) for 5 days. BAT *Gpr3* gene expression was determined by RT-qPCR. Data are presented as fold change (n = 3 per group, males). ∗∗P ≤ 0.05, ∗∗P ≤ 0.01, ∗∗∗P ≤ 0.001. ∗∗∗∗P ≤ 0.0001.

Strikingly, rescue of either ERRα or ERRγ restored cold-inducibility of *Gpr3* transcription in AdipoQ-Cre *Esrra/g* KO mice (Figure 4A). We assessed gene and protein expression of *Ucp1*/UCP1 to compare the transcriptional regulation of *Gpr3* to another cold-induced ERRα/γ target [38]. While the transcriptional response of *Ucp1* to cold was dramatic, the protein induction was more modest, in line with previous reports of 24 h cold exposure [56,57]. Levels of *Ucp1* were reduced in the AdipoQ-Cre *Esrra/g* KO mice at either temperature, but the cold-inducibility of *Ucp1*/UCP1 was not dependent on the ERRs (Figure 4A–B). Our observations are in line with previous cold exposure studies of ERRα whole-body KO mice [36,37].

Creatine kinase B (CKB) regulates thermogenesis by driving the futile creatine cycle, a parallel pathway to UCP1-dependent thermogenesis [58]. As with *Gpr3*, it has previously been established that *Ckb* is transcriptionally cold-induced by the ERRs [48]. Additionally, BAT-specific GPR3 overexpression itself enhances *Ckb* mRNA levels [48]. Since there is currently no GPR3 antibody available, we used CKB as a proxy to validate the functional rescue at the protein level. Cold-inducibility of CKB was rescued by AAV-mediated delivery of either ERRα or ERRγ (Fig. 4B). Interestingly, the CKB rescue was more pronounced in the mice that had been infected with ERRγ AAVs (Fig. 4B), which could, similar to the luciferase assay, be explained by a less PGC-1α-dependent activity of this ERR isoform [40,54,55].

As further downstream characterization, we measured expression of a number of genes known to be activated by cAMP signaling or involved in adipose thermogenesis. Interestingly, we noted that these genes had differential responses. We observed that the cold-inducibility of *Atf3*, *Cidea*, *Prdm16* and *Cox7a1* was dependent on the presence of ERRα or ERRγ, yet the expression of *Dio2*, *Elovl3* and *Ppargc1a* was not (Fig. S2).

Finally, we investigated the potential to pharmacologically target the ERRs to induce expression of *Gpr3 in vivo*. Indeed, when we treated wild type mice with the pan-ERR agonist SLU-PP-915 [44], we observed a significant induction of *Gpr3* expression in BAT (Fig. 4C).

## 4 Discussion

The ERRs are orphan nuclear receptor transcription factors with no known endogenous ligand. The ligand binding pocket of ERRα was initially thought to be very restricted based on a crystal structure of the ligand binding domain complexed with PGC-1α [30]. However, Kallen et al. uncovered the potential for multiple dramatic conformational changes in the ligand binding pocket of ERRα, which supports the possibility of a bulkier endogenous ligand [59]. It is tempting to speculate that a lipolysis-regulated metabolite would provide the missing link between lipolysis and the role of these receptors in the transcriptional control of *Gpr3*. Of note, the major adipocyte lipase, hormone sensitive lipase (HSL), is highly active in metabolism of both diglycerides and cholesteryl esters [60]. An alternative means by which lipolysis may influence ERRα/γ activity is via regulation of the ERR coactivators, PGC-1α and PGC-1β. This is supported by the ChIP-qPCR results presented here, which indicate that ERRα is bound to the *Gpr3* promoter even in the “off” state (when the gene is not transcribed). In skeletal muscle, NR corepressor 1 (NCoR1) represses ERRα under basal conditions. However, during stimulated conditions, NCoR1 exchanges for PGC-1α which promotes transcription [61]. Therefore, it may be the lipolysis-induced exchange or binding of a coactivator that triggers the “opening” of the region and subsequent transcriptional induction of *Gpr3*.

Of note, the work presented here does not preclude the possibility of lipolysis-dependent elements and enhancers, located further upstream or downstream of the cold-sensitive promoter. Indeed, the differential transcription profiles of *Ucp1* and *Gpr3* in cold exposed AdipoQ-Cre *Esrra/g* KO mice suggest a more complex transcriptional mechanism of BAT ERRα/γ. While ERRα/γ were required for cold induction of *Gpr3* and *Ckb*/CKB, they were dispensable for *Ucp1/*UCP1 induction. Other downstream targets of Gα_s_ and cAMP signaling showed this same differential pattern. CKB and UCP1 are known to act in parallel pathways to regulate cold-induced thermogenesis in adipocytes through non-paralogous and complementary pathways [62]. Our evidence aligns with previous reports that UCP1-independent mechanisms of thermogenesis (such as the futile creatine cycle) are transcriptionally dependent on ERRα/γ, whereas UCP1 can be induced independently of ERRα/γ [63]. Yet, this does not disqualify the need for ERRα/γ in activation of thermogenesis downstream of UCP1.

Finally, while ERRα/γ inverse agonism displays benefits in models of diabetes via hepatic engagement [28,64,65], agonists targeting the ERRs in muscle and BAT may also improve metabolic outputs. In skeletal muscle, ERRα/γ enhanced oxidative capacity [34,35,51,54,[66], [67], [68]], and Mootha et al. have proposed that ERRα agonism may offset the molecular implications of skeletal muscle insulin resistance in patients living with type 2 diabetes [42]. In BAT, ERRα/γ mediate the adaptive response to adrenergic stimulation [38], and our results show that the pan-ERR agonist SLU-PP-915 activates *Gpr3* transcription. In line with this, administration of an earlier synthetic pan-ERR agonist, SLU-PP-332, reduce obesity and improve insulin sensitivity in mouse models of metabolic syndrome [69]. We, therefore, propose that ERR agonism may provide a therapeutic strategy for harnessing GPR3-induced calorie-burning in thermogenic fat.

## 5 Conclusions

In conclusion, the cold-induced transcription of *Gpr3* in brown fat is dependent on ERRα/γ, which bind to a cold-sensitive element in the promoter of *Gpr3*. Whether this transcriptional axis directly mediates the lipolysis-dependent element of *Gpr3* transcription remains a subject for further investigation.

## CRediT authorship contribution statement

**Olivia Sveidahl Johansen:** Writing – original draft, Visualization, Investigation, Conceptualization. **Rebecca L. McIntyre:** Writing – review & editing, Writing – original draft, Visualization, Investigation. **Janane F. Rahbani:** Writing – review & editing, Investigation. **Qiaoqiao Zhang:** Writing – review & editing, Investigation. **Charlotte Scholtes:** Writing – review & editing, Investigation. **Damien Marc Lagarde:** Writing – review & editing, Investigation. **Cyrielle Billon:** Writing – review & editing, Investigation. **Isabelle Côté:** Writing – review & editing, Investigation. **Maria Delgado-Martin:** Writing – review & editing, Investigation. **David Tandio:** Writing – review & editing, Investigation. **Astrid Linde Basse:** Writing – review & editing, Investigation. **Elodie Eury:** Writing – review & editing, Investigation. **Anastasia Kralli:** Writing – review & editing, Investigation. **Thomas P. Burris:** Writing – review & editing, Investigation. **Vincent Giguère:** Writing – review & editing, Investigation. **Lawrence Kazak:** Writing – review & editing, Investigation, Conceptualization. **Zachary Gerhart-Hines:** Writing – review & editing, Writing – original draft, Investigation, Conceptualization.

## Data and materials availability

Data are available upon request. Plasmids and mouse models used in this study will be available upon request.

## Declaration of competing interest

The authors declare the following financial interests/personal relationships which may be considered as potential competing interests: Zachary Gerhart-Hines reports financial support was provided by Novo Nordisk Foundation. Zachary Gerhart-Hines reports financial support was provided by European Research Council. Rebecca McIntyre reports financial support was provided by European Molecular Biology Organization. Anastasia Kralli reports financial support was provided by the National Institutes of Health. Vincent Giguere reports financial support was provided by Canadian Institutes of Health Research. Vincent Giguere reports financial support was provided by Terry Fox Research Institute. Lawrence Kazak reports financial support was provided by Canadian Institutes of Health Research. Zachary Gerhart-Hines reports a relationship with Embark Laboratories ApS that includes: employment. Zachary Gerhart-Hines reports a relationship with Incipiam Pharma ApS that includes: employment. Olivia Sveidahl Johansen reports a relationship with Embark Laboratories ApS that includes: employment. If there are other authors, they declare that they have no known competing financial interests or personal relationships that could have appeared to influence the work reported in this paper.

## Data Availability

Data will be made available on request.
