# Supplementary material for: Cold exposure induces the constitutively active thermogenic receptor, GPR3, via ERRα and ERRγ
Source: Mol Metab. 2025 Oct 30;103:102277. doi: 10.1016/j.molmet.2025.102277 (PMC12639564; doi:10.1016/j.molmet.2025.102277)
Supplement: Multimedia component 1 [file mmc1.docx]

**Supplementary**

**
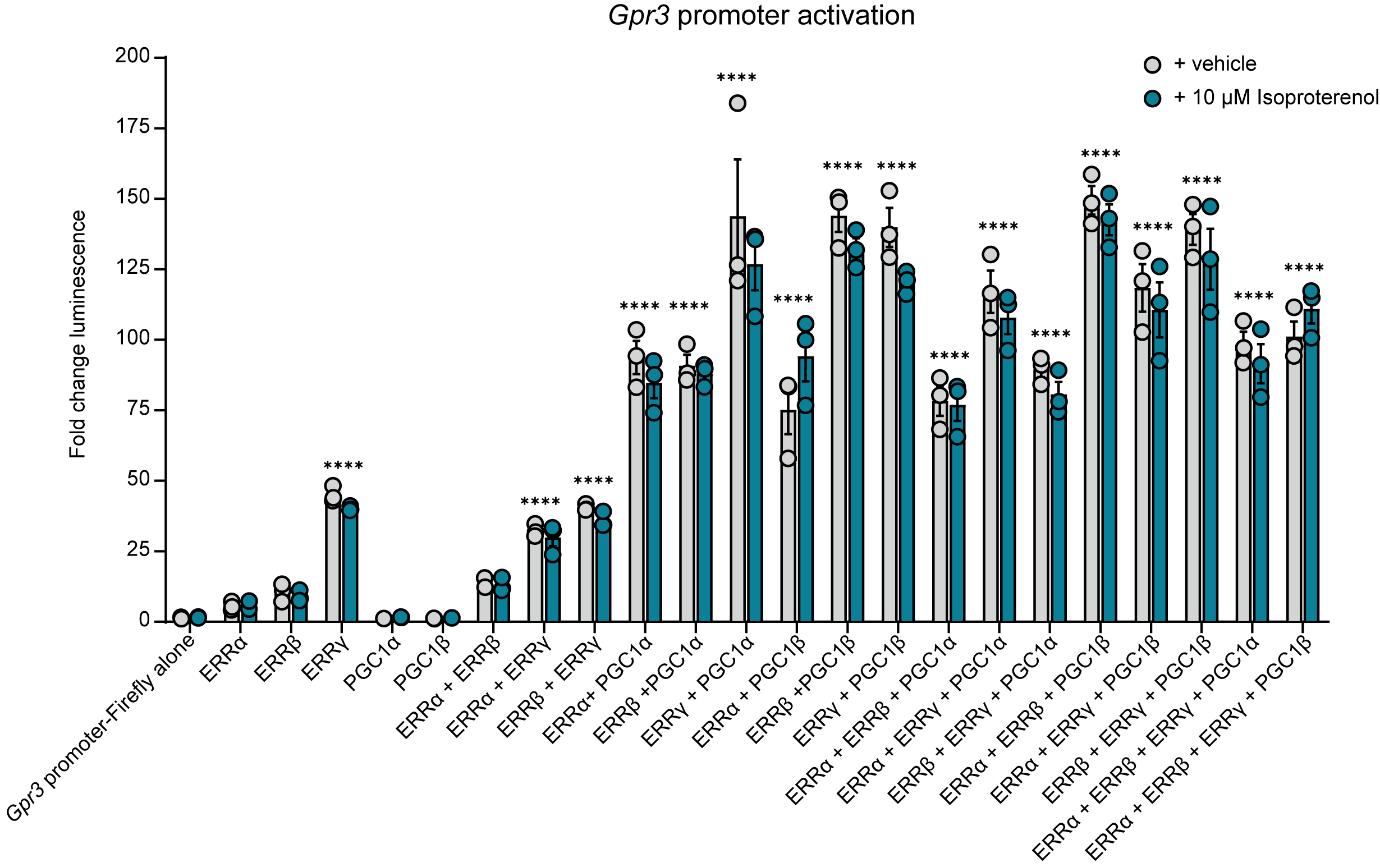
**

Figure S1: Co-transfection of combinations of the ERRs and PGC1s activate the *Gpr3* promoter

HEK293T cells were co-transfected with a firefly luciferase reporter plasmid linked to the Gpr3 promoter and plasmids expressing the indicated pcDNA expression vectors, along with a NanoLuc control plasmid for normalization. At 24 h post-transfection, cells were treated with empty media (vehicle) or media containing 10 µM isoproterenol. Luciferase activity was assayed 5 h later, and firefly values were normalized to NanoLuc luminescence. Activity is expressed relative to the reporter plus an empty pcDNA vector. Data were analyzed by two-way ANOVA; post hoc comparison using Šídák’s multiple comparisons test comparing each transfection condition to transfection with the *Gpr3* promoter-Firefly construct alone. There was no measurable statistical difference in luminescence when comparing the effect of isoproterenol within a transfection condition. Data are shown as the mean + SEM; ****P ≤ 0.0001.


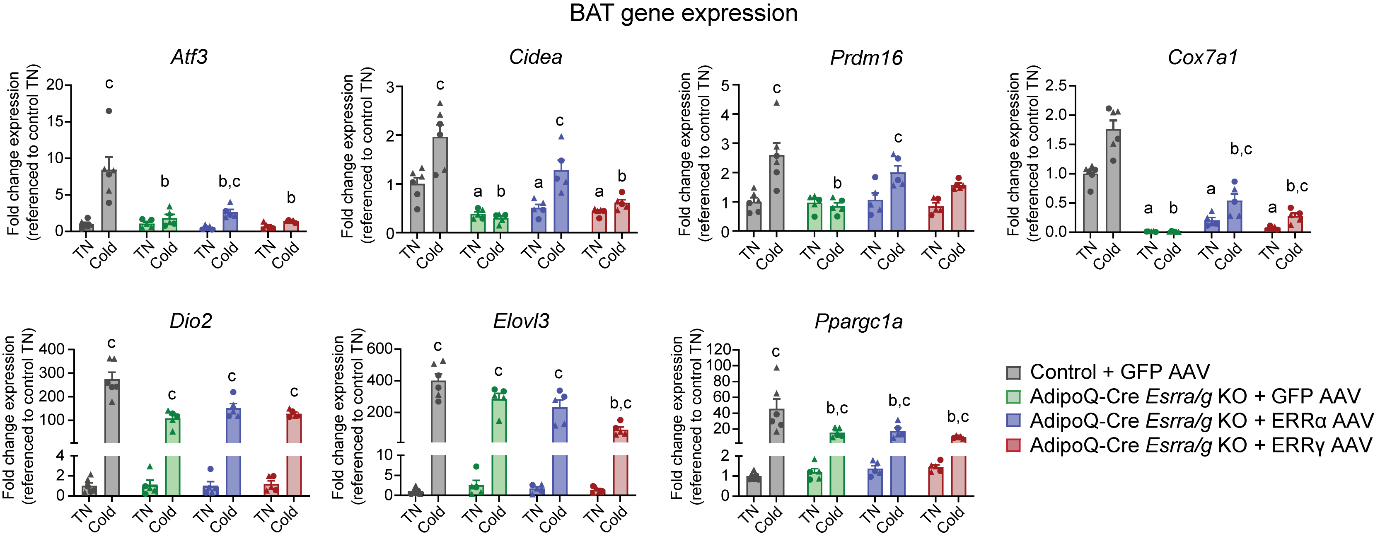


Figure S2: Differential responses of thermogenic genes to AAV-mediated rescue of ERRα and ERRγ

RT-qPCR measuring gene expression of genes known to be responsive to Gα_s_ signaling or cAMP from BAT isolated from mice treated as in Figure 3A. RT–qPCR data were log_10_ transformed prior to analysis by two-way ANOVA; post hoc comparison between control groups and AdipoQ-Cre *Esrra/g* KO groups and between temperatures within each genotype using Šídák’s multiple comparisons test. a=at TN, significantly different from control group. b=at cold, significantly different from control group, c=significantly different between TN and cold within group. Data presented as fold change. (n = 5-6 per group, females (circles) and males (triangles)).

Supplementary table 1

Reagents:

| **Reagent** | **Company** | **Cat#** |
| --- | --- | --- |
| 293T | ATCC | - |
| DMEM | Wisent | 319-005-CL |
| FBS | Multicell | 098150 |
| Penicillin/streptomycin | Multicell | 450-201-EL |
| TransIT-X2 Dynamic Delivery System | Mirus Bio | MIR6005 |
| Opti-MEM reduced serum medium | Gibco | 31985 |
| Nano-Glo Dual-Luciferase Reporter Assay System | Promega | N1630 |
| Carprofen | Zoetis Canada | Rimadyl injectable solution |
| Lidocain | Vetoquinol | Lurocaine |
| Bupivacaine | Sensorcaine | Bupivacaine Injection BP |
| Polyethylenimine (1 mg/ml) | Polysciences | 23966-1 |
| AAVanced Concentration Reagent | System Biosciences | AAV100A-1 |
| AAV helper plasmid pDP8 | Plasmid Factory | PF421-180518 |
| Low-fat diet | Envigo | 2920X |
| Chow diet (2018 Teklad Global 18% Protein Rodent Diets) | Inotiv | - |
| SLU-PP-915 | [44] | - |
| Trizol | Invitrogen | 15596026 |
| High-Capacity cDNA Reverse Transcription kit | ThermoFisher Scientific | 4368814 |
| SYBR-green PCR master mix kit | ThermoFisher Scientific | 4309155 |
| High-Capacity cDNA Reverse Transcription kit | Applied Biosystems | 4368813 |
| QIAzol | Qiagen | 79306 |
| GoTaq qPCR Master Mix | Promega | A6001 |
| cOmplete™ Protease Inhibitor Cocktail | Roche | 11836170001 |
| BCA assay | Pierce | 23225 |
| 4x Laemmli protein sample buffer | Bio-Rad | 161-0747 |
| Clarity | Bio-Rad | 1705060 |
| Dynabeads protein G | Thermo Fisher Scientific | 10009D |
| SYBR Green I Master Mix | Roche | 4887352001 |

Supplementary table 2

Antibodies:

| **Antigen** | **Company** | **Cat#** | **Dilution** |
| --- | --- | --- | --- |
| VCL | Cell signaling | 13901 | 1:5000 |
| CKB | Abcam | ab151579 | 1:1000 |
| UCP1 | Abcam | ab10983 | 1:2000 |
| ERRα | Abcam | ab76228 | 1:1000 |
| GFP | Abcam | ab290 | 1:2500 |
| Anti-rabbit | Promega | W401B | 1:10000 |

Supplementary table 3

Primers:

| **Gene** | **Forward primer** | **Reverse primer** |
| --- | --- | --- |
| *36b4* | TCATCCAGCAGGTGTTTGACA | GGCACCGAGGCAACAGTT |
| *Ucp1* | AAGCTGTGCGATGTCCATGT | AAGCCACAAACCCTTTGAAAA |
| *Gpr3* | ATCACCTGAGCAACCGAGAA | AGATGGGGGTGCATTTTACA |
| *Ckb* | GCCTCACTCAGATCGAAACTC | GGCATGTGAGGATGTAGCCC |
| *Gfp* | AAGGGCATCGACTTCAAGG | TGCTTGTCGGCCATGATATAG |
| *Esrra* | CCAGAGGTGGACCCTTTGCCTTTC | CACCAGCAGATGCGACACCAGAG |
| *Esrrg* | CTCCAGCACCATCGTAGAGGATC | GATCTCACATTCATTCGTGGCTG |
| *Atf3* | GCCCCTGAAGAAGATGAGAGG | CTGACTCTTTCTGCAGGCACT |
| *Cidea* | GTCAAAGCCACGATGTACGA | CAGGAACTGTCCCGTCATCT |
| *Prdm16* | CCTGTGGAGTCCTGAAAGA | CAGCTTCTCCGTCATGGTTT |
| *Cox7a1* | AAAACCGTGTGGCAGAGAAG | CAGCGTCATGGTCAGTCTGT |
| *Dio2* | GCTTCCTCCTAGATGCCTACAA | AGTCAAGAAGGTGGCATTCG |
| *Elovl3* | ATGAATTTCTCACGCGGGTT | AGCTTACCCAGTACTCCTCCA |
| *Ppargc1a* | TGATGTGAATGACTTGGATACAGACA | GCTCATTGTTGTACTGGTTGGATATG |
| ChIP *Gpr3* primer set 1 | CATAGTAATGAGGCCTCGCGCC | TAGTGTCCCCAAAACCCCCGAC |
| ChIP *Gpr3* primer set 2 | GGGAGGTCACAGAGGATGTGGG | CAGCTCGTTTCTGCCACCGAAG |
| *Esrra* -3722  neg. | TTGGCATTGATATTGGGGGTGGGAGCAACT | GACTTCTTACTTTGACGCTTTCCTCCATCG |
| *Prox1* -55772 (ctrl) neg. | CCAAGCACAAATATCTAATCACCCTTTC | CTTCTTGATAGGTTTATGGGTTGGGC |
| *Essra* (WT and conditional alleles) | CCCTGCTTCTGTGCCCTTTGC | CCACCACTGCCCAGCTTCAC |
| *Essrg* (WT and conditional alleles) | GTTTTAAAGGCCCTTGGT GATCTCGC | CTGCAACCCTTGGACTGCCAGAAC |
| *AdipoQ-Cre* | GACATGTTCAGGGATCGCCAGGCG | GACGGAAATCCATCGCTCGACCAG |
